# The effect of particle size on drug bioavailability in various parts of the body

**DOI:** 10.1016/j.pscia.2023.100031

**Published:** 2023-11-23

**Authors:** Zi Hong Mok

**Affiliations:** Swansea University Medical School, Swansea, UK

**Keywords:** Particle size, Nanoparticles, Microparticles, Absorption, Internalisation

## Abstract

Multiple mechanisms are involved in driving the efficacy of drug delivery. Drug particle size is one of the challenges as particles need to be delivered from the external environment, into the circulation or interstitial fluid and transiting the cell membranes for cellular internalisation. Small particles are presumably easier to be internalised, yet they are not easy to retain as they are subject to fast clearance. Big particles do not cross biological barriers as easily, but their size distribution is easier to be controlled. Because of the various routes of administration, the size range of these particles will also need to be catered for the anatomical, biological, and dynamic barriers involved. This review hopes to provide an insight into the range of particle size that has been engineered for drug delivery via various routes of administration of the body, such as to cross the epithelium of gastrointestinal tract, lungs, skin, blood-brain barrier, kidney and liver, the eye, nose, and ear, the cancer tumour matrix and into the muscles. While successful drug delivery also depends on the material properties of the delivery systems and the bio/nano interface related properties, this review focuses on the importance of particle size for enhancing bioavailability at the various organs of the body.

## 1 Introduction

Turning a new chemical entity (NCE) into an effective medication is a challenge. Active pharmaceutical ingredient needs to be delivered to where it is intended to be for it to exert its pharmacological action. This requires the control of many physicochemical parameters, including particle size, porosity, degree of crystallinity, and biochemical properties, such as chemical interactions of the drug/carrier with biomolecules. The pure drug substance needs to be formulated into a dosage form, which then breaks back up into particles. They would then need to overcome the biological barriers, subsequently dissolve for absorption, or remain as small particles for uptake by internalisation, whilst bypassing the clearance either by the reticuloendothelial system or the kidneys.

In these various stages of drug formulation and delivery, particle size matters because it informs whether the drug is suitable to be formulated into the required dosage form, its stability profile, and whether it is small enough to overcome the biological barriers for it to be absorbed or internalised. Smaller particles of a bulk drug have a larger specific surface area to allow for interfacial solubility. A larger specific surface area also allows for adhesion and interactions with cell membranes for cellular uptake. Ultimately, these affect the rate and extent of absorption of drugs, which are paramount for making the drugs bioavailable to the human body. Therefore, multiple approaches have been adopted for reducing drug particle size, for example, milling and micronisation of pharmaceutical powders, making microemulsions, entrapping drugs in micro or nanoparticles, and precipitating drugs into micro or nanocrystals. These are especially useful for the absorption of poorly soluble drugs, as a drug first needs to be solubilised prior to absorption or be in the nano size range to maximise the chance for internalisation.

Whilst there are many rate-limiting steps in drug absorption, in relation to the physicochemical properties of the drug, particle size is of utmost importance. Since particle size is usually well-characterised in research papers, this manuscript aims to collect the research and review how drug particle size affects the drug bioavailability at various parts of the body. In this review, an update of recent research on overcoming biological barriers in relation to particle size of the drug or the carrier, including the epithelium of gastrointestinal tract, lungs, skin and blood-brain barrier, kidney and liver, and administrations into the eye, nose and ear, the cancer tumour matrix and intramuscular vaccine is reviewed.

## 2 Drug delivery at various parts of the body

The efficacy of a drug is primarily dependent on its successful delivery into the body. Furthermore, drugs intended to have site-specific therapeutic actions will need to have the right physicochemical properties to be delivered to the local part of the body. The intrinsic characteristics of the drug or the material properties of the delivery systems and the bio/nano interface related properties will affect whether the drug or carrier can be absorbed or internalised. Focusing on the particle size of the drug or delivery system as one of the major determinants, a summary of the relevant literature for drug delivery at various parts of the body is provided below.

### 2.1 Gastrointestinal drug delivery

**Fig. 1:**
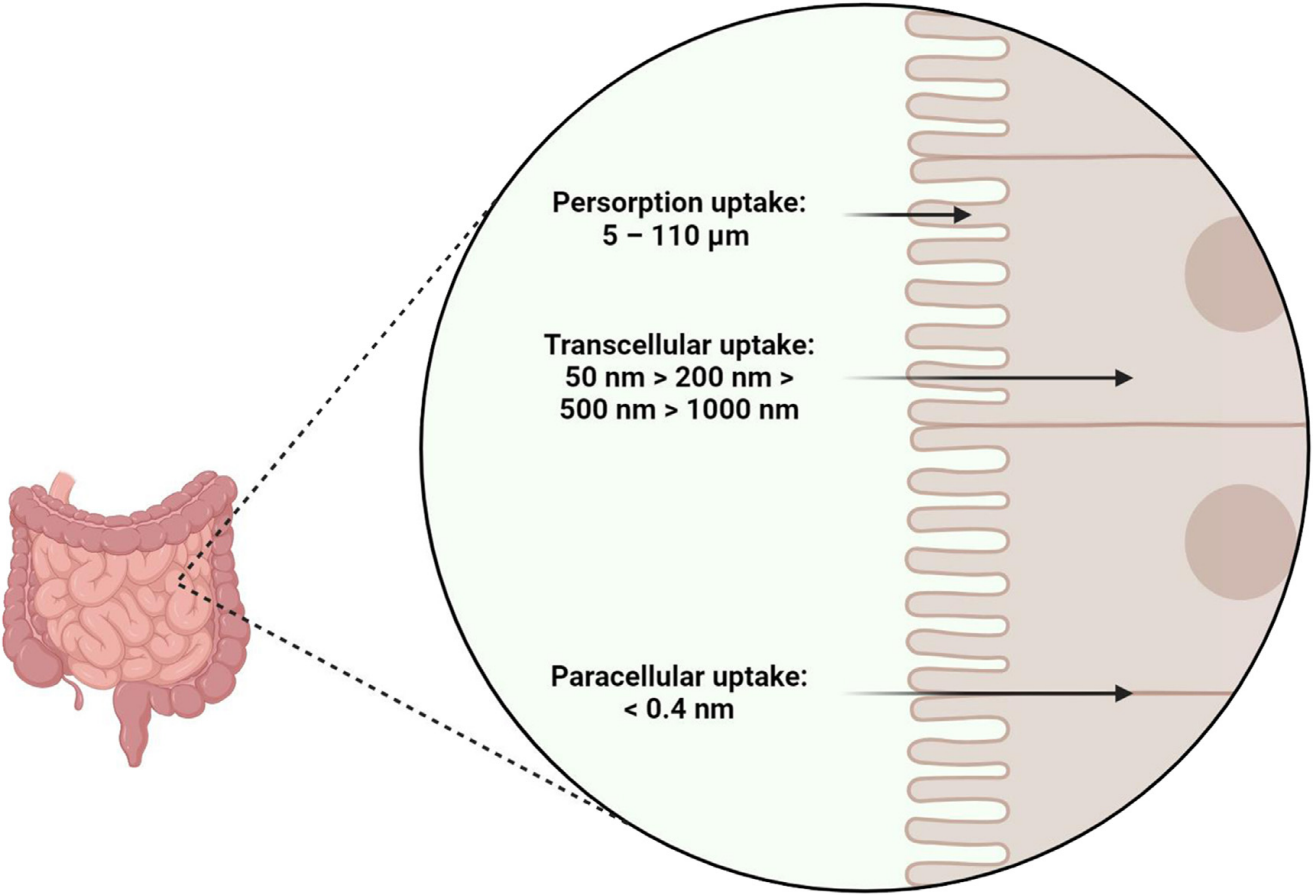
Particle size required for gastrointestinal drug delivery. Persorption may occur with undissolved particles between 5 and 110 μm. There is an inverse correlation with particle size for transcellular uptake. Meanwhile, the pores of the tight junctions have a cut-off of 0.2 nm radius for paracellular uptake.

Drug carriers with a particle size greater than 5 μm are hardly absorbed in the intestinal tract [1]. For particles smaller than that, absorption can occur via the routes of transcellular (through a cell) or paracellular (between the cells) (see Fig. 1).

For transcellular uptake, there is an inverse correlation with particle size (transcellular uptake: 50 nm > 200 nm > 500 nm > 1000 nm) [2]. Meanwhile, bigger particles will need to dissolve prior to absorption. For example, with the proton pump inhibitor drug esomeprazole, the smallest particle size has shown the fastest drug dissolution (dissolution: 494 μm > 648 μm > 1400–2000 μm) [3]. The dissolution of carvedilol nanosuspension (212 nm) is also greater than its microsuspension (4895 nm) and much coarser suspension, which has also enhanced its absorption [4]. The reduction of drug particle size promotes the diffusion of the drug across the unstirred water layer, the brush border membrane of enterocytes, and the interfacial interaction with the mucin layer [4]. For passive transport like this, the higher the lipophilicity of the drug, the greater is the diffusion, as the drug needs to partition through the lipid bilayer of the cells. Dissolved particles undergo passive diffusion if the molecular weight of the substance is less than 450 Da [5].

Membrane permeability is typically limited when the polar surface area of the drug exceeds 14 nm^2^ [6], thus, hydrophilic drugs can diffuse via the paracellular route; however, this is limited by the tight junctions, as pores of the tight junctions have a cut-off of 0.2 nm radius [7]. Therefore, larger hydrophilic drugs will have to penetrate the cells via the transmembrane proteins using receptor-mediated transport of the transcellular pathway. Active transport typically is needed for dissolved molecules and particles up to 200 nm. It is worth noting that the use of surfactants, such as sodium dodecyl sulphate in nanoparticle formulations can “exfoliate” the intestinal epithelium due to its potential lytic nature and cause structural separation of the tight junctions to enhance paracellular absorption [8].

Although happening in low quantities, persorption can occur, which is when undissolved particles (15 – 75 μm [9] or 5 – 110 μm [10]) get kneaded into the mucous of the digestive tract, pass through the epithelial cells into the subepithelial layer through breaks in the tips of the villi, get transported into the blood circulation via the lymph vessels and the mesenteric veins. Cytopempsis, a form of micropinocytosis by the epithelial cells lining the intestine, can also uptake particles with a diameter up to 50 μm [9]. However, the low frequencies of these mechanisms make them unlikely to be exploited for the delivery of therapeutic agents, hence the lack of recent research into these mechanisms.

### 2.2 Respiratory drug delivery

Heyder has previously reported on how inhaled particles of different sizes are deposited in the respiratory tract. Carried by tidal air through the respiratory system, inhaled particles are subject to mechanical forces such as gravity, inertia, and gas molecule collisions [11]. Particles smaller than 0.1 μm in diameter are entirely deposited by diffusion, as the distance a particle moves by diffusion increases with smaller particle size [11]. Diffusion of particles to the alveolar space enables soluble particles to dissolve in the alveolar surface fluid, diffuse through the epithelium into the lymph or blood. Particles in the size range of 0.1–1 μm are simultaneously deposited by diffusion and sedimentation, as gravitational deposition increases with larger particle size [11]. Sedimentation controls deposition in the lower bronchial airways and the gas exchange area. Particles greater than 1 μm are deposited by sedimentation and impaction, as inertial transport is effective for larger particles [11]. Impaction causes the deposition in the extrathoracic and upper bronchial airways. All inhaled particles smaller than 10 μm in diameter have the potential of being biologically active in the respiratory system [11]. It is also important to note that inhaled hydrophilic particles can expand from water vapour uptake from surrounding moist air, therefore changing the deposition distribution [11].

**Fig. 2:**
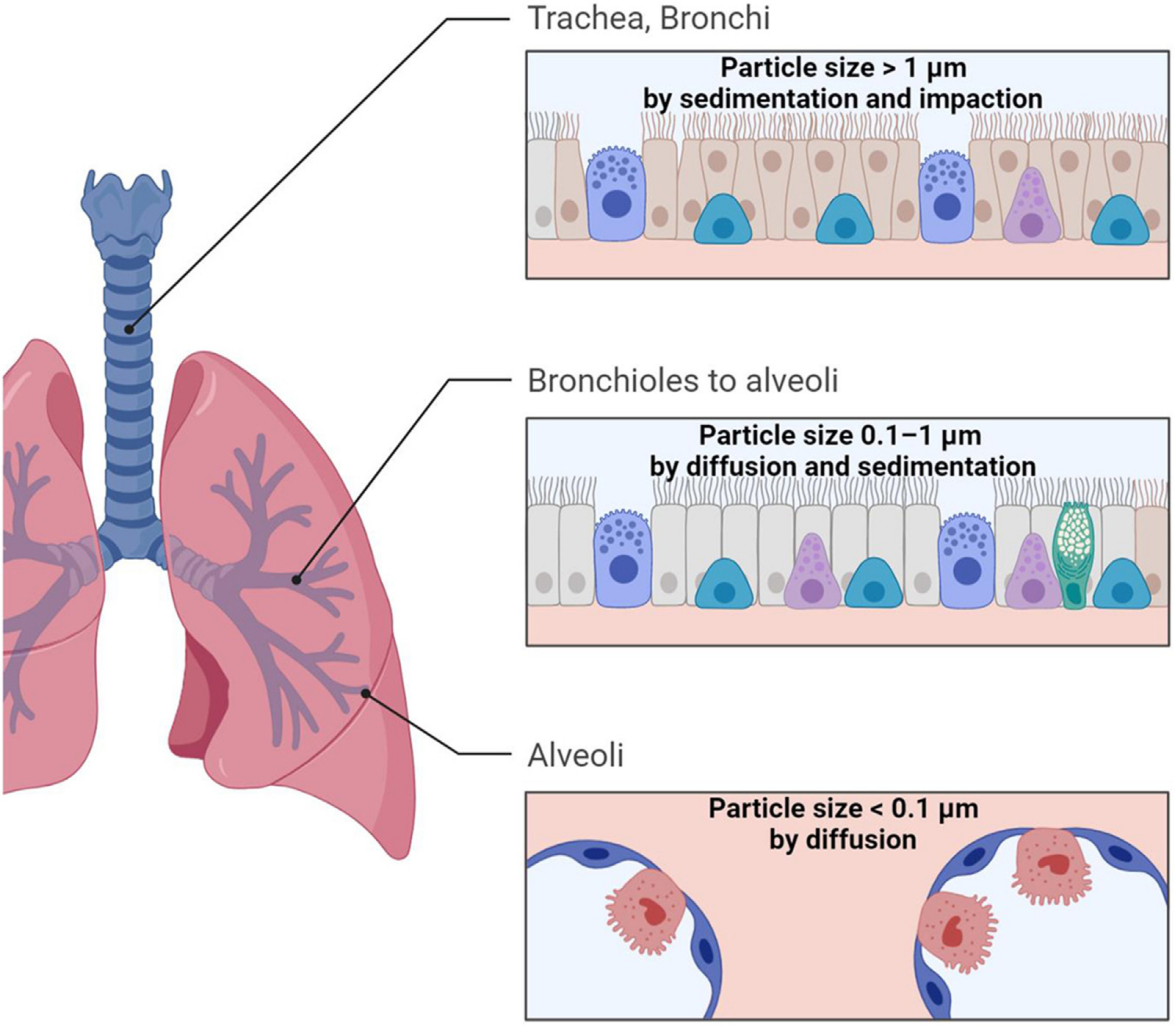
Particle size required for respiratory drug delivery. Particles smaller than 0.1 μm in diameter are entirely deposited by diffusion. Particles in the size range of 0.1–1 μm are simultaneously deposited by diffusion and sedimentation. Particles greater than 1 μm are deposited by sedimentation and impaction.

A region is effectively targeted with a drug if more than 50% of the drug delivered to the respiratory tract deposited in that area [11]. Drug particle size between 1 and 5 μm is needed for entry into the deep lung by inhalation and particles of 1–2 μm are most suitable for reaching the small airways (an important anatomical target for the treatment of asthma and COPD) and alveolar epithelium (an important target for systemic delivery/absorption of orally inhaled products) (see Fig. 2) [12]. Small, monodispersed salbutamol particles (1.5 μm) achieved significantly better total lung deposition and lung penetration than larger particles (6 μm) [13]. With inhaled corticosteroids, there is also an increased deposition in the distal lung or small airways (beyond conducting airways) with smaller particles. However, it is worth noting that this does not appear to translate into improved clinical outcomes for patients with asthma, as firstly deposition at the conducting airways is also required, and secondly there is only a transient effect on narrowed airways despite finer aerosols depositing there [14]. Inhaled drug particles are also readily trapped by the mucous gel layer and rapidly cleared from the lung via the mucociliary clearance, continuous ciliary beating of the periciliary layer (underlying the mucous gel layer), or cough-mediated expectoration, thereby reducing the residence time for them to be internalised [15].

Apart from particle size, targeting lung regions requires control of breathing parameters (slow and steady for metered dose inhalers; quick and deep for dry powder inhalers). Particles of 6 μm with 10 s of breath-holding can be used to achieve complete deposition in small airways; particles of 1 μm with breath-holding can target the small peripheral lung structures for the topical treatment of peripheral respiratory diseases, or even the potential of the drug to be delivered to the blood circulation [11].

### 2.3 Skin drug delivery

**Fig. 3:**
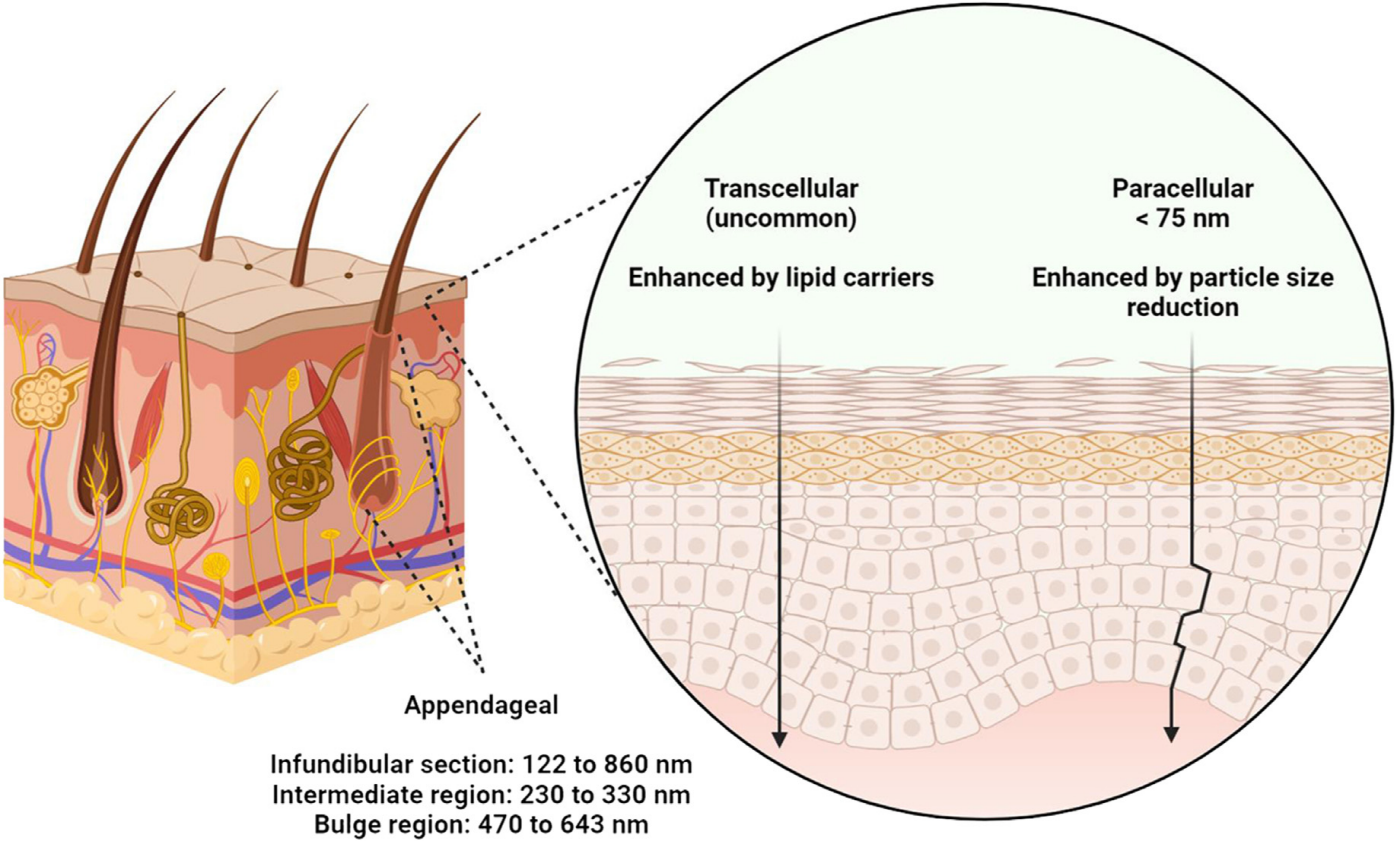
Particle size required for skin drug delivery. Transcellular route remains uncommon for drug permeation through the skin, while the paracellular route has a gap of 75 nm. Hair follicles have also been explored as a pathway for drug delivery for nanoparticles.

Transport of molecules can occur through the skin via the transcellular, paracellular and appendageal (such as hair follicles, sweat glands, and sebaceous glands) routes. The stratum corneum acts as a physical barrier, due to keratin filaments within a filaggrin matrix retained by the corneocytes, and the cornified lipid wrapping that takes over the cell membrane of the keratinocyte [16]. Because of the barrier crossing both lipophilic and hydrophilic structures, transcellular route remains uncommon for drug permeation through the skin [17]. Meanwhile, the paracellular route has a gap of 75 nm [18] and will allow non-polar molecules with a molecular weight lesser than 500 Da and log P 1–4 to diffuse via this route [19]. (see Fig. 3).

Given the challenges involved in transporting substances across the skin, lipid-based colloid carriers which have structural similarities with those composing epidermis have been used to enhance drug permeation through the skin. They could attach themselves onto the skin surface, promote adhesiveness and increase skin hydration, gradually lead to detached skin structure through polarity alteration, fluidisation, and lipid exchange within the intercellular lipid domain [20]. For example, liposomes with mean diameters between 31 and 41 nm have exhibited significantly enhanced penetration through the skin [21]. Particle size reduction also helps with skin penetration, as NSAIDs (indomethacin, ketoprofen and piroxicam) incorporated into nanoparticles of 70–90 nm (with zirconia beads and hydroxypropyl cellulose) has enhanced percutaneous penetration compared to their normal formulation [22]. The accumulation of poly(l-lactide-co-glycolide) nanoparticles of 70 nm at the inflammation site of dermatitis models is also greater than that of 300 nm [23].

Hair follicles remain a pathway for diffusion into systemic circulation, as they, including hair roots and sebaceous glands, represent around 0.1% of the skin surface, with thin stratum corneum layer in the deeper parts of hair follicles, and the invagination that facilitates access to the capillary network [19]. Nanoparticles of approximately 600 nm in diameter have been shown to penetrate the hair follicles with a skin massage, as the moving hair pushes the nanoparticles into the hair follicles [24]. The infundibular section (upper portion of the follicle) can be targeted with particles of 122 to 860 nm, intermediate region with particles of 230 to 330 nm, and the bulge region with particles of 470 to 643 nm [25]. Solid lipid nanoparticles less than 100 nm has also shown the most encouraging skin penetration rate and depth mainly via hair follicles [26].

### 2.4 Brain drug delivery

The blood-brain barrier (BBB) is the interface between the blood (luminal) and the brain (abluminal), whereby there is a tight regulation of movement of ions, molecules and cells by the blood vessels that vascularise the central nervous system (CNS) for neuronal functions. The endothelial cells are held together by tight junctions, limiting the paracellular uptake [27]. There is also a low prevalence of transcellular transport across BBB that remains unexplored, including (macro)pinocytosis, clathrin-dependent and caveolin-dependent endocytosis, and the subsequent trafficking of vesicles to the opposite membrane [28].

**Fig. 4:**
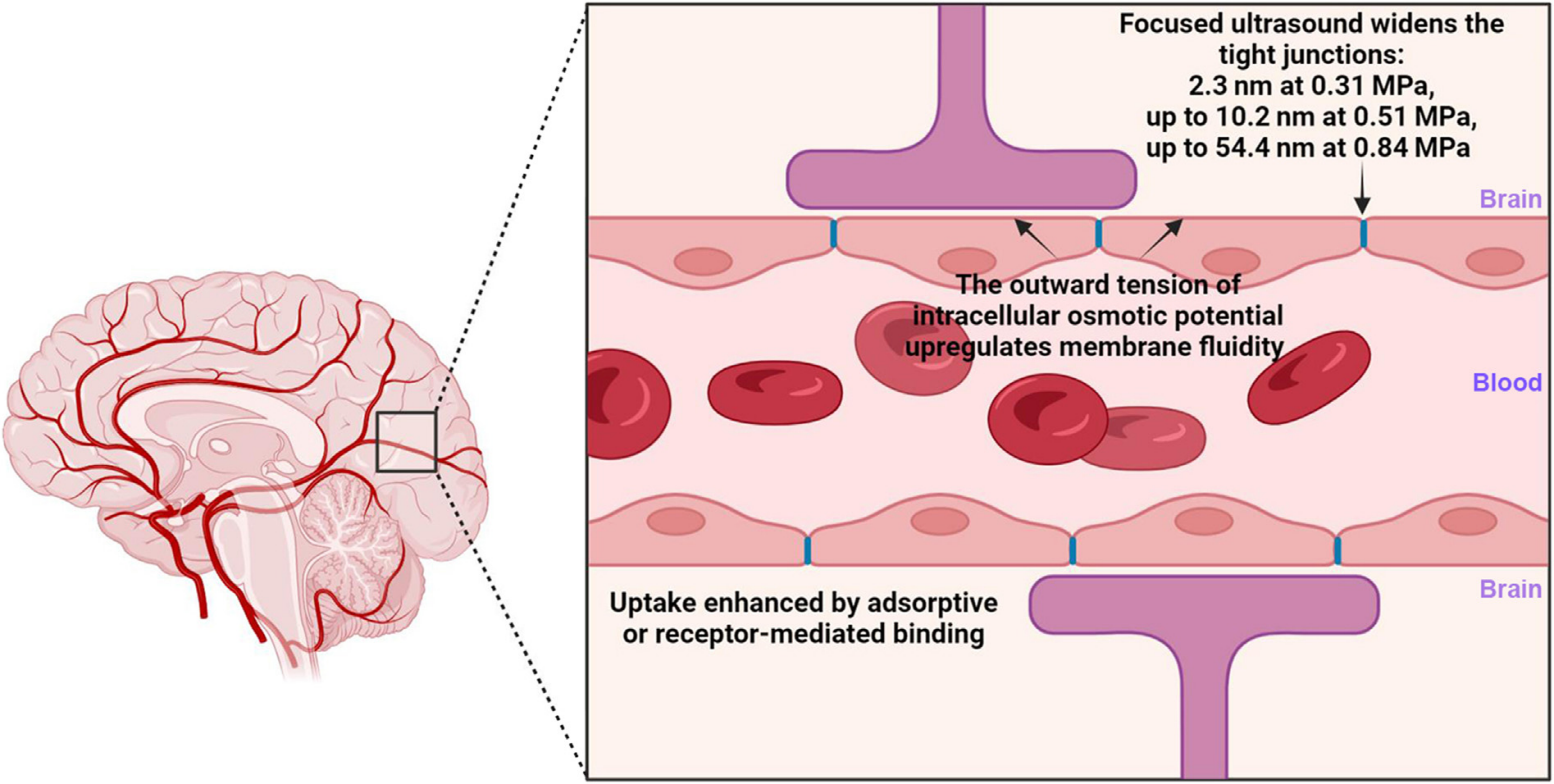
Particle size required for brain drug delivery. The endothelial cells are held together by tight junctions, limiting the paracellular uptake through the BBB. Approaches to promote BBB leakage for drug delivery purposes have been carried out, such as by using focused ultrasound or by increasing the intracellular osmotic pressure.

Approaches to promote BBB leakage for drug delivery purposes have been carried out. Enhanced uptake of nanoparticles through the BBB can be induced by focused ultrasound (FUS). FUS induces inertial or stable cavitation with microbubbles that exerts a mechanical force onto capillary walls, leading to a temporary opening of the BBB via the widening of tight junctions [29]. It was found that the BBB opening size was smaller than 3 kDa (2.3 nm) at 0.31 MPa, up to 70 kDa (10.2 nm) at 0.51 MPa, and up to 2000 kDa (54.4 nm) at 0.84 MPa [30]. Gold nanoparticles of 3 and 15 nm have been delivered into the brain assisted by FUS-induced BBB opening [29]. The membrane fluidity of the BBB can also be enhanced by an increase in the intracellular osmotic pressure (see Fig. 4). For example, the depolymerisation of cytoskeletons (microfilament and microtubule) due to extracellular BBB lesion causes an increased production of intracellular protein nanoparticles (such as actin and α/β-tubulin) [31]. They carry negative charges that would adsorb cytoplasmic cations (such as K^+^) thereby inducing extracellular cation (mainly Na^+^) influx [31]. The accumulation of cations causes a charge gradient, leading to the influx of negative ions and eventually intracellular hyperosmolarity. The outward tension of intracellular osmotic potential upregulates membrane fluidity and could promote non-selective drug influx [31]. Similarly, by infusing a hypertonic solution of arabinose or mannitol into the carotid artery, the tight junctions can be widened by endothelial cell shrinkage, vascular dilatation associated with removal of water from the brain, and modulation of the contractile state of the endothelial cytoskeleton and junctional proteins by increased intracellular calcium [32].

Despite the low rate of transcytosis that restricts vesicle-mediated transcellular uptake, nanoparticles have been shown to penetrate the BBB transcellularly via adsorptive (positively charged nanoparticles to negatively charged endothelial cell plasma membrane) or receptor-mediated (ligands on nanoparticles binding to targets such as GLUT1, lactoferrin or transferrin or peptides such as angiopep-2 or Seq12) approaches [33]. For example, by targeting the BBB insulin receptors, insulin-coated gold nanoparticles of 20 nm have shown the highest accumulation within the brain as compared to 50 and 70 nm particles of the same nature [34]. Carbamazepine to treat epilepsy, being loaded in methoxy poly(lactide-co-glycolide)-b-poly(ethylene glycol) methyl ether (mPEG–PLGA) nanoparticles, also showed the highest accumulation in the brain for 60 nm particles compared to 90 and 120 nm particles [35]. As demonstrated, on top of particle size, it is important to note that particle composition also play a role in the penetration of the tightly regulated BBB [36].

### 2.5 Cancer tumour drug delivery

**Fig. 5:**
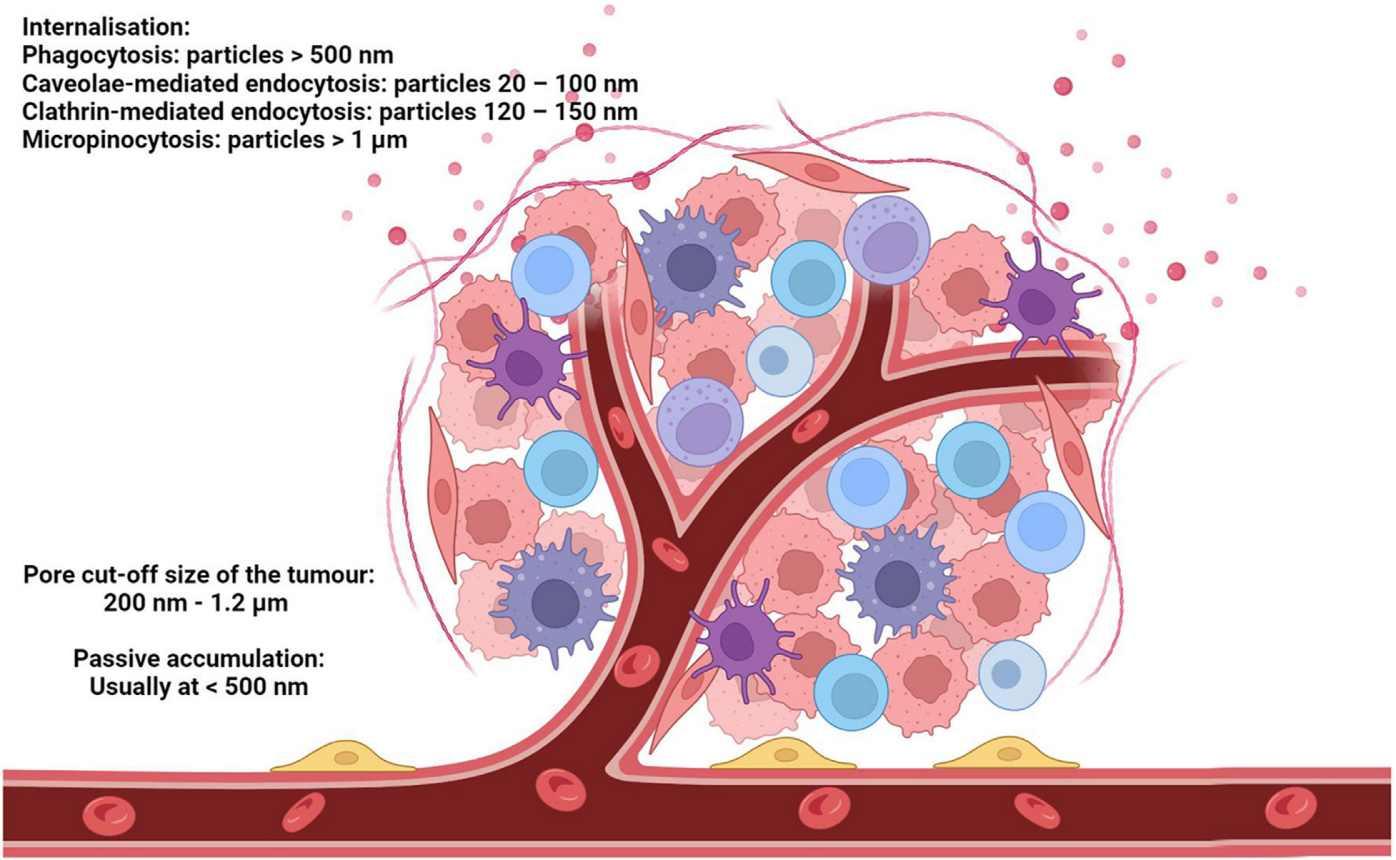
Particle size required for cancer tumour drug delivery. In general, nanoparticles of size <500 nm from the blood circulation can passively accumulate in the tumour cells due to EPR effect.

Hypervascularisation (increased number of blood vessels), extravasation (leaky vasculature) from blood vessels to tumour tissues and impaired lymphatic drainage contribute to the enhanced permeability and retention (EPR) effect. Depending on the tumour type and microenvironment, the pore cut-off size of the tumour vessel ranges from 200 nm to 1.2 μm [37]. In general, nanoparticles of size <500 nm from the blood circulation can passively accumulate in the tumour cells [38] (see Fig. 5). Intratumorally, the smaller the nanoparticles, the faster the diffusion [39] and the deeper into the dense extracellular matrix of solid tumours [40]. For example, only the 30 nm micelles, compared to 50, 70 and 100 nm, could penetrate poorly permeable pancreatic tumours to achieve an antitumour effect [41]. However, smaller nanoparticles are subject to quicker clearance from the tumour (due to the high interstitial fluid pressure of the tumour) and through the kidneys. For example, 50-nm drug–silica nanoconjugates outperforms its smaller (20 nm, due to fast clearance from tumours) and larger (200 nm; due to of limited tumour tissue penetration) analogues in overall tumour tissue accumulation and retention [42]. Internal (enzymes, pH, and redox) and external (light and temperature) stimuli have also been introduced to change the morphology of the original nanodrugs as size-tunable strategies for tumour targeted delivery [43].

Cellular uptake or internalisation of the nanoparticles after permeation and retention within the tumour tissues are also dependent on the particle size. This can happen via phagocytosis (particles >500 nm) or pinocytosis (caveolae-mediated endocytosis: particles 20 – 100 nm; clathrin-mediated endocytosis: particles 120 – 150 nm; micropinocytosis: particles >1 μm) [44]. Intracellular retention is further enhanced by decreased exocytosis.

### 2.6 Kidney and liver

Fenestration of the peritubular capillary (small blood vessels in the kidney) is sized 70–90 nm, and the glomerular filtration barrier (GFB) in a healthy kidney has slits at about 6–10 nm [45]. Subsequently, nanoparticles larger than 6–10 nm cannot penetrate the GFB of the kidneys, thus avoiding clearance by the kidneys [46]. On the other hand, nanoparticles sized less than 10 nm or proteins smaller than 20 kDa can distribute at kidney tubules, but they are less likely to retain in the body. Nanoparticles with negative surface charges are more likely to be blocked out by the filtration barrier because the GFB is negatively charged. In diseased state however, both the size and charge control of the barrier can be impaired, allowing particles with larger size passing through.

**Fig. 6:**
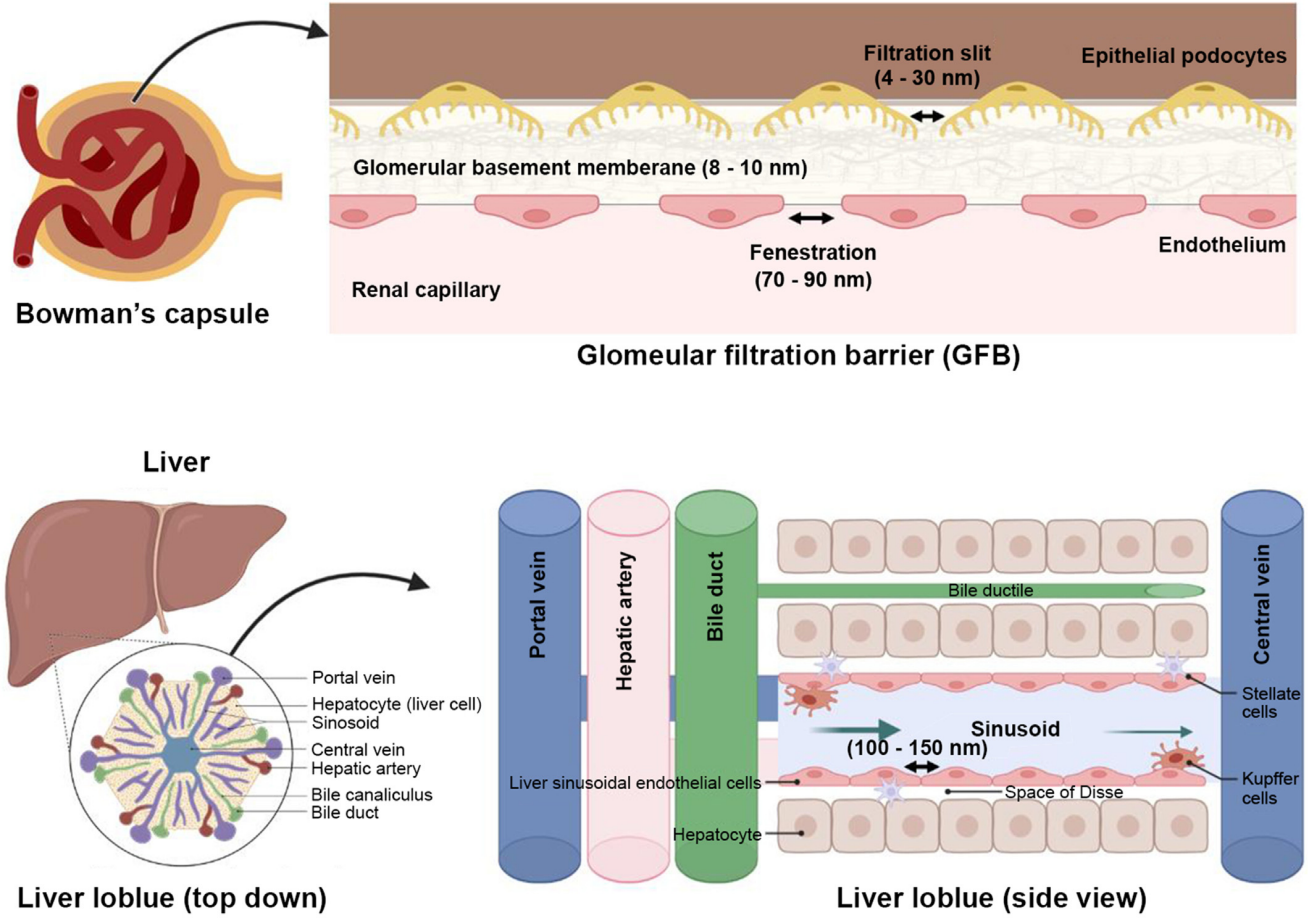
Particle size required for kidney and liver drug delivery. Nanoparticles larger than 10 nm cannot penetrate the GFB of the kidneys, thus avoiding clearance by the kidneys. Meanwhile, particles which bypass RES clearance and have size approximately 100–150 nm in diameter may pass through the liver sinusoidal fenestrae.

Nanoparticles that reach the liver can undergo clearance either by biliary excretion (endocytosis and enzymatic breakdown by hepatocytes → bile → gut → excretion) or phagocytosis (Kupffer cells in the reticuloendothelial system/RES). Particles which bypass RES clearance and have size approximately 100–150 nm in diameter may pass through the liver sinusoidal fenestrae [47] (see Fig. 6). Nanoparticles such as liposomes, micelles, exosomes, polymeric nanoparticles including chitosan, dendrimers, albumin nanoparticles, metallic, silica and carbon nanoparticles have been explored for targeted delivery both to the kidneys and liver.

### 2.7 Intramuscular drug delivery

Medications given by the intramuscular route include biologicals (such as vaccines), antibiotics (such as cephalosporins), and hormones (such as testosterone or oestradiol). This is because muscles have high vascularity for drug to be absorbed relatively rapidly into the blood without first-pass metabolism. Intramuscular route is preferred when drugs are not suitable to be given subcutaneously due to stinging irritation, or when a quicker absorption is required due to greater blood supply to the large bulk muscles. Intramuscular route is also preferred when a slightly longer drug release is preferred over the intravenous route.

Most vaccine delivery systems are particulate (including nanoparticles, microparticles or adjuvant-formulated proteins), with effective size range of 20–200 nm [48]. Lipid nanoparticles have successfully been introduced to deliver mRNA to fight against Covid-19. Decorated by positively charged lipids, the mRNA (negatively charged) is more stable and resistant to nuclease degradation. Once injected intramuscularly, antigen-presenting cells (including neutrophils, monocytes, macrophages, and dendritic cells) take up lipid nanoparticles at both the injection site and in draining lymph nodes [49]. The size of lipid nanoparticles should be large enough to recruit immune cells, yet small enough for the uptake by these cells to generate antibodies [50]. Particle size ranging between 75 and 95 nm in diameter have shown to be most efficacious in mRNA expression and immunogenicity [51]. This is because particles of this size (within 20–200 nm) can preferentially be taken up by dendritic cells [52]. These nanoparticles are internalised mainly via clathrin- and caveolae-mediated endocytosis (same mechanism as the SARs-CoV-2 virus which is 100 nm in size [53]). Clathrin prefers orchestrating the uptake of small particles (<100 nm in diameter) due to the size constrains of the clathrin lattice [54]. That said, tetanus toxoid with aluminium adjuvants which is taken up via clathrin-mediated endocytosis by nerve cells at the neuromuscular junctions, has a wide particle size range of 0.5–4000 μm [55].

The subsequent endosome entrapment then requires endosomal escape to deliver the mRNA to ribosomes to be translated for transfection efficiency [56]. Lipid nanoparticles (positively charged) is drawn to the inner membrane of the endosomes (negatively charged), enabling fusion to allow the translocation of mRNA to the cytoplasmic water phase outside the endosomes [57]. The mRNA would then dissociate from the lipid nanoparticles, giving rise to intracellularly available mRNA for protein synthesis [57]. A proportion of non-dissociated mRNA-lipid could take part in the invagination of the endosomal membrane to form intraluminal vesicles packaged with mRNA [57]. These vesicles are then released into the extracellular environment upon the fusion of multivesicular bodies with the plasma membrane and are released outside the cell as extracellular vesicles, taken up by other cells for the mRNA to be released there [57].

Meanwhile, particles between 500 and 5000 nm are preferentially taken up by macrophages by phagocytosis [52]. Phagocytosis is different to endocytosis, as it does not rely on coat proteins (such as clathrin and caveolin) but is mediated by the actin cytoskeleton to wrap larger particles [54].

### 2.8 Eye, nose, and ear drug delivery

Polymer microspheres (spherical microscopic particles that range in size from 1 to 1000 μm) are highly researched for drug delivery into the eyes. This is because they can encase more than one active substances, the dose can be adjusted for personalised medicines, and the concentrations can be maintained within the eyes. Drugs that can be encased include antiproliferative, anti-inflammatories, immunosuppressant, antibiotics, and biological therapeutic agents. These drugs can be given via routes such as topical, systemic through posterior end, periocular (surrounding the eyeball) or intraocular such as intravitreal (fluid behind lens) and intracameral (anterior chamber).

For subconjunctival (beneath the conjunctiva) injection of nanoparticles and microparticles, it was found that 20 nm polystyrene particles could cross the sclera to a minor degree, whereas 200 nm and 2 μm particles could not and were retained in the periocular tissues [58,59]. Smaller particles (20–100 nm) can more easily pervade scleral tissues through the sieving of sclera's collagen fibres [60]. The spacing of collagen fibre bundles in the sclera is around 300 nm [61]. This anatomical barrier also affects drug delivery into the suprachoroidal space (circumferential space in between the sclera and choroid around the eye) for drug delivery to the back of the eye, as particles must be able to permeate through a portion of the sclera to reach the suprachoroidal space.

Other barriers encountered for drug delivery to the eyes include static barriers (corneal layers, sclera, retina, blood aqueous and retinal barriers), dynamic barriers (tear dilution, conjunctival and choroidal blood flow, lymphatic clearance), and efflux pumps [62]. Despite subjecting to dilution, the bioavailability of suspended drug depends on the retention and dissolution of drug particles in the tear fluid. It was demonstrated that small particles of indomethacin (median size: 0.37–1.33 μm) yielded higher concentrations of dissolved indomethacin in the tear fluid compared to larger particles (median size: 3.12–3.50 μm), thereby leading to improved ocular bioavailability [63].

Microspheres as a carrier can overcome rapid nasal mucociliary clearance (which reduces the residence time of drugs) and increase drug absorption. The ideal microsphere particle size requirement for nasal delivery should range from 10 to 50 μm as smaller particles than this will enter the lungs [64]. For mometasone furoate suspended particles, the size range of 1–5 μm did not affect the total nasal epithelial uptake, but smaller particles are able to dissolve more quickly than larger ones, resulting in a faster initial uptake and minimises the chance for the drugs deposited at the back of the throat to be swallowed [65].

Mucoadhesion is also important for nasal drug delivery. Chitosan microspheres for example can enhance the paracellular absorption. The interaction of the positively charged chitosan (amino groups) with negatively charged mucus (sialic acid residues) enables mucoadhesion [66], and with negatively charged cell membranes induces a structural reorganisation of tight junction-associated proteins such as ZO-1 [67].

**Fig. 7:**
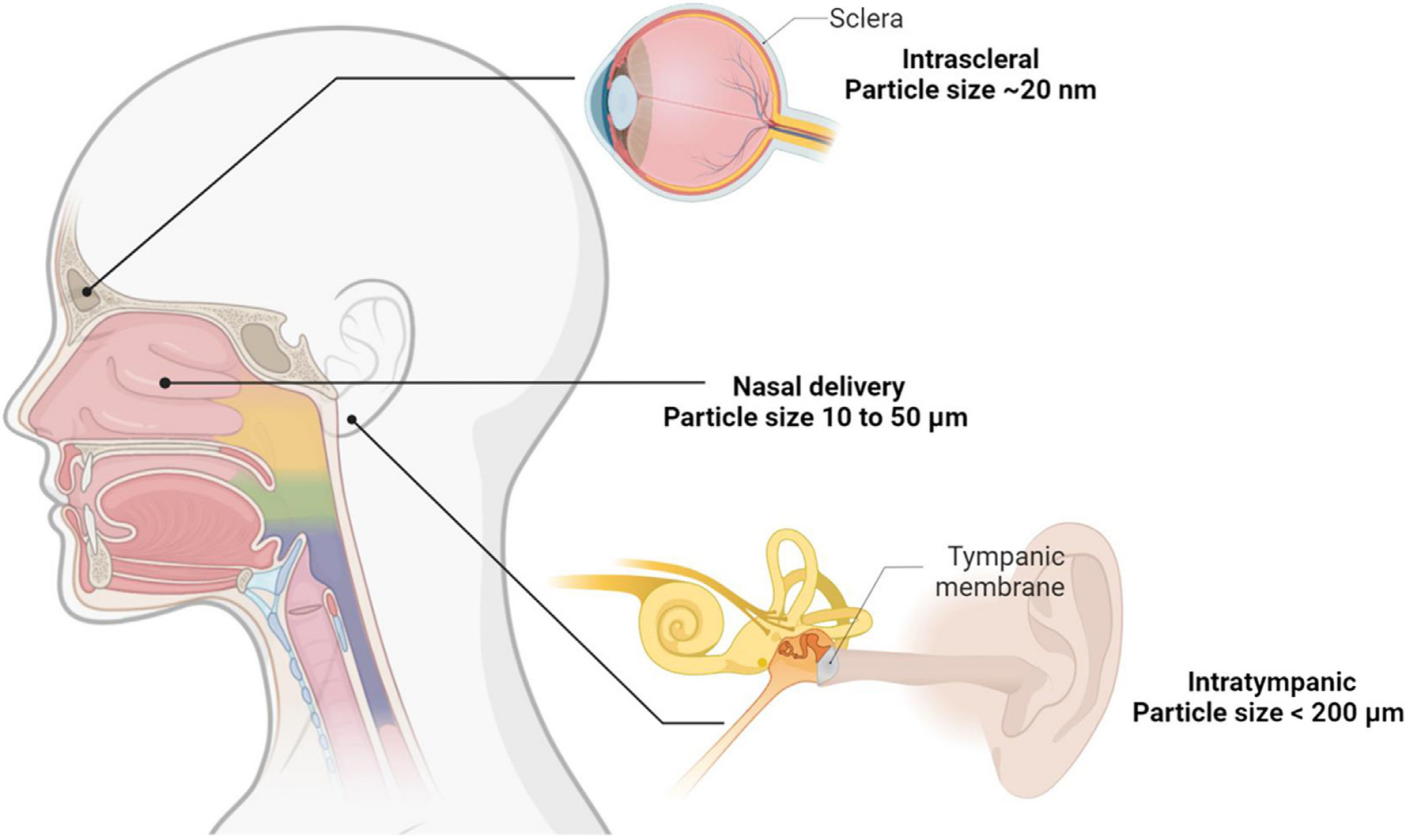
Particle size required for eye, nose, and ear drug delivery. 20 nm polystyrene particles could cross the sclera to a minor degree. The ideal microsphere particle size requirement for nasal delivery should range from 10 to 50 μm. Nanoparticles less than 200 nm can carry drugs into the inner ear by crossing the round window membrane.

By injecting formulations into the middle ear (intratympanic), nanoparticles less than 200 nm can carry drugs into the inner ear by crossing the round window membrane (one of the two openings from the middle ear into the inner ear) [68] (see Fig. 7). Of three sizes of liposome nanoparticles (95, 130 and 240 nm), the highest transport is the 95 nm liposomes, and it decreases with increasing size [69]. Computational simulations also suggest that decreasing particle sizes (2000, 200 and 20 nm) contribute to higher diffusion rate toward the contralateral ear via the Eustachian tube [70]. Premature elimination of drug particles through the Eustachian tube may be reduced by formulating the particles in a hydrogel [71]. A summary of all literature reviewed in this article on overcoming biological barriers in relation to particle size of the drug or the carrier is provided in Table 1.

**Table 1:** Comparison of literature findings on the effect of particle size on drug bioavailability in various parts of the body. Other physicochemical properties of the drugs or carriers would also have an effect, but not exclusively discussed here.

No.	Target parts	Main findings	References
1	Stomach and intestines	An inverse correlation with particle size for transcellular uptake.	[2]
		Hydrophilic drugs can diffuse via the paracellular route limited by the tight junctions with pores having a cut-off of 0.2 nm radius.	[7]
		Persorption can occur when undissolved particles of 5 – 110 μm get kneaded into the mucous of the digestive tract.	[10]
2	Lungs	Drug particle size between 1 and 5 μm is needed for entry into the deep lung by inhalation and particles of 1–2 μm are most suitable for reaching the small airways and alveolar epithelium.	[12]
3	Skin	Transcellular route remains uncommon, whereas the paracellular route has a gap of 75 nm and will allow non-polar molecules with a molecular weight lesser than 500 Da and log P 1–4 to diffuse.	[18,19]
4	Brain	FUS leads to temporary opening of the BBB via the widening of tight junctions, with an opening size smaller than 3 kDa (2.3 nm) at 0.31 MPa, up to 70 kDa (10.2 nm) at 0.51 MPa, and up to 2000 kDa (54.4 nm) at 0.84 MPa.	[30]
5	Cancer tumour	Nanoparticles of size <500 nm from the blood circulation can passively accumulate in the tumour cells through the EPR effect. Various other active targeting mechanisms have been used.	[38]
6	Kidneys	Nanoparticles larger than 10 nm cannot penetrate the GFB of the kidneys, thus avoiding clearance by the kidneys.	[46]
7	Liver	Particles which bypass RES clearance and have size approximately 100–150 nm in diameter may pass through the liver sinusoidal fenestrae.	[47]
8	Muscles	Most vaccine delivery systems (nanoparticles, microparticles or adjuvant-formulated proteins) have an effective size range of 20–200 nm.	[48]
9	Eyes	20 nm polystyrene particles could cross the sclera to a minor degree.	[58]
10	Nose	The ideal microsphere particle size requirement for nasal delivery should range from 10 to 50 μm.	[64]
11	Ears	Nanoparticles less than 200 nm can carry drugs into the inner ear by crossing the round window membrane.	[68]

## 3 Conclusions and perspective

Knowing the particle size of the drug and its carrier, amongst multiple complex internalisation mechanisms, is a prerequisite of successful drug formulation and delivery. Particle sizes in pharmaceutics include small drug particles or carriers in μm or nm, from passively targeting its site of action, to nanoparticle encapsulation and surface modification for optimal active uptake. Many studies have demonstrated the right particle size for processing solid dosage forms and the travel pathways of drug particles. Their particle size also determines their ability to cross the biological barriers for optimal drug delivery. The particle size reviewed here offers an insight into the range required for these applications, especially looking into the common routes of administration including the gastrointestinal tract, lungs, skin, kidney, liver, eye, nose, and ear, and into the blood-brain barrier, the cancer tumour matrix, and intramuscular administrations. It is prudent to note that successful drug delivery will also depend on the material properties of the delivery systems and the bio/nano interface. However, serving as an important attribute in pharmaceutics, the more is known about particle size and how it affects the particle behaviour and target application, the higher is the chance for optimal drug formulation and delivery. Accurate particle size determination and report are therefore important for establishing the effect of particle size on drug bioavailability at various targets of the body.

## Author contributions

ZHM performed the literature review and analysis, drafted, revised, and improved the paper. The author has read and approved the final manuscript.

## Data availability

Not applicable.

## Ethics approval

Not applicable.

## Funding

This research received no funding.

## Declaration of competing interest

The authors declare that they have no known competing financial interests or personal relationships that could have appeared to influence the work reported in this paper.
